# Rough microsomes isolated from snap-frozen canine pancreatic tissue retain their co-translational translocation functionality

**DOI:** 10.1093/biomethods/bpaf044

**Published:** 2025-06-02

**Authors:** Marianne Croonenborghs, Marijke Verhaegen, Eva Pauwels, Becky Provinciael, Kurt Vermeire

**Affiliations:** Molecular, Structural and Translational Virology, Department of Microbiology, Immunology and Transplantation, Rega Institute, KU Leuven, 3000 Leuven, Belgium; Molecular, Structural and Translational Virology, Department of Microbiology, Immunology and Transplantation, Rega Institute, KU Leuven, 3000 Leuven, Belgium; Molecular, Structural and Translational Virology, Department of Microbiology, Immunology and Transplantation, Rega Institute, KU Leuven, 3000 Leuven, Belgium; Molecular, Structural and Translational Virology, Department of Microbiology, Immunology and Transplantation, Rega Institute, KU Leuven, 3000 Leuven, Belgium; Molecular, Structural and Translational Virology, Department of Microbiology, Immunology and Transplantation, Rega Institute, KU Leuven, 3000 Leuven, Belgium

**Keywords:** protein translocation, canine pancreatic microsomes, endoplasmic reticulum, co-translational translocation, Sec61 translocon, cell-free in vitro protein translation

## Abstract

Proteins are essential for life in all organisms: they mediate cell signaling and cell division and provide structure/motility to cells and tissues. All proteins are synthesized on cytoplasmic ribosomes as unfolded precursors that need to find their correct location in the compartmentalized cell. In eukaryotes, ∼30% of the proteome is translocated across or integrated into the endoplasmic reticulum (ER) membrane, a process mostly mediated by the heterotrimeric Sec61 complex that spans the ER membrane. There is significant interest in identifying small-molecule inhibitors of the Sec61 translocon channel that hold great promise as putative anticancer, immunosuppressive, or antiviral drugs. Hence, representative models are needed to study Sec61-dependent protein import into the ER. Microsomal membranes (or microsomes) isolated from dog pancreatic tissue are the primary source of mammalian ER for cell-free *in vitro* protein translocation research. Here, we demonstrate that for the isolation of microsomal membranes, snap-frozen canine pancreatic tissue can serve as a valuable alternative to freshly isolated organ tissue from euthanized animals. For 17 out of 20 animals, a sufficient yield of microsomes was extracted from defrosted pancreatic tissue. The isolated microsomes contained the essential proteins of the translocation machinery, and proved to be intact as verified by the detection of ER lumenal chaperones. Importantly, 13 out of the 17 microsome samples retained their translocation competence, as reflected by successful *in vitro* co-translational translocation of wild-type bovine preprolactin. The microsomes supported post-translational modifications of the tested substrates such as signal peptide cleavage and N-linked glycosylation. Furthermore, the tested microsome samples responded well to the translocation inhibitor cyclotriazadisulfonamide in suppressing human CD4 protein translocation into the ER. In conclusion, microsomes isolated from frozen canine pancreatic tissue proved to retain their co-translational translocation functionality that can contribute to our research of Sec61-dependent protein translocation and selective inhibition thereof.

## Introduction

All proteins are synthesized on cytoplasmic ribosomes as unfolded precursors that need to find their correct location in the compartmentalized cell. Protein co-translational translocation into the endoplasmic reticulum (ER) is an essential process in cells, as it represents a crucial step in the biogenesis of secretory and integral membrane proteins, delivered in the extracellular environment or integrated in the cell membrane, respectively [1]. During this process, the translating ribosome protein complex is targeted from the cytosol to the ER membrane where the nascent polypeptide chain is translocated via a protein-conducting channel, the Sec61 translocon, into the ER lumen.

Nascent polypeptide chains of client proteins are targeted to the Sec61 translocon by intrinsic hydrophobic targeting signals, i.e. signal peptide (SP) or internal transmembrane domains (TMDs) [2]. When this targeting signal emerges from the ribosome during protein translation, it is recognized by a cytosolic signal recognition particle (SRP) [3]. SRP also directs the ribosome nascent chain complex to the ER membrane by binding to its membrane-bound receptor. Subsequently, the nascent chain is transferred in a GTP-dependent manner from SRP to the Sec61 translocon and its targeting signal induces conformational changes in the channel such as the displacement of the plug domain and repositioning of the lateral gate helices toward the opened conformation [1]. As structural models have advanced in explaining the dynamic interactions of the Sec61 translocon upon protein insertion, it has become evident that the hydrophobic strength of the targeting signal is essential for successful protein translocation [4, 5]. Proteins with weaker hydrophobic SPs and/or TMDs depend on accessory components for efficient translocation, such as translocon-associated protein (TRAP), translocating chain-associated membrane protein (TRAM) and binding immunoglobulin protein (BiP) [5]. During co-translational protein translocation N-terminal SPs are removed from the preprotein by signal peptidases located in the ER lumen [6]. Finally, for their proper folding and function, mature proteins are post-translationally modified in the ER, such as N-glycosylated by the lumenal oligosaccharyl transferase (OST) complex [7].

The Sec61 translocon, which is highly conserved across all eukaryotes, is a dynamic protein-conducting channel consisting of three subunits: Sec61α, Sec61β, and Sec61γ. The α-subunit is composed of 10 transmembrane helices (TMHs) embedded in the ER membrane and forms the central pore of the channel through which nascent peptide chains pass to reach the ER lumen. In mammals, Sec61α is encoded by two genes, Sec61A1 and Sec61A2, with Sec61A2 being the less abundantly expressed isoform [8]. Sec61β is a single-membrane spanning protein that makes only limited interactions with the periphery of the α-subunit and has been considered dispensable for channel functioning [1, 9]. However, Sec61β has been shown to kinetically favor co-translational translocation of clients [10]. Sec61α contains two covalently linked pseudo-symmetric halves, formed by TMHs 1–5 and TMHs 6–10, and is clamped by the smaller peripheral Sec61γ subunit. The lumenal side of the translocon is closed off by a plug domain (TMH2a) that can be displaced to allow vertical protein transport along the Sec61 translocon. In addition, TMHs 2 and 7 of Sec61α function as the lateral gate, facilitating lateral insertion of hydrophobic TMDs of integral membrane proteins into the lipid bilayer of the ER membrane [5, 11].

Cell-free translation systems from rabbit reticulocyte lysate (RRL) are commonly used to study protein biogenesis *in vitro*. However, proper translocation and processing of secretory and membrane proteins during translation require the presence of ER organelles. Thus, ER-derived membranes isolated from pancreatic tissue have been widely used in the research field of mammalian protein translocation. Moreover, rough microsomes isolated from canine pancreatic tissue are often the primary source for *in vitro* translocation studies as these microsomes exert the best translocation efficiency and give the most reproducible results [12–16]. Although alternatives using yeast or insect cell systems have been developed to reduce reliance on animal sources, the dog pancreas microsome system remains a model standard due to its high compatibility and established protocols for accurate *in vitro* protein translocation and membrane integration research [17–20]. As mentioned above, in order to support successful co-translational protein translocation, ER-derived membranes must contain translocon chaperones and several active enzymes, such as signal peptidase and OST. Therefore, extraction of rough microsomes is generally performed on a freshly isolated pancreas from euthanized animals, making access to this tissue rather challenging. Here, we present that pancreatic tissue that has been snap-frozen can serve as a valuable alternative to fresh material for the extraction of ER-derived microsomes that preserve translocation competence.

## Materials and methods

### Compound

Cyclotriazadisulfonamide (CADA) hydrochloride was purchased from TCG Lifesciences Pvt. Ltd (Kolkata, India), who synthesized the compound as described previously [21]. CADA was dissolved in dimethyl sulfoxide (DMSO) and stored at a stock concentration of 10 mM at room temperature.

### Plasmids and mutagenesis

The pGEM4 expression vector encoding bovine preprolactin (pPL) was kindly provided by Prof. Kalies (University of Lübeck, Lübeck, Germany). The pcDNA3 expression vector encoding wild-type full-length human CD4 was a kind gift from Prof. Schwartz (Institut Pasteur, Paris, France). Truncated human CD4 D1D2 was generated via PCR as described earlier [22]. The sequence encoding the signal peptide and 11 amino acid residues of the mature domain of CD86 was fused to the N-terminus of mature prolactin in a pGEM4 vector via molecular cloning. Briefly, inserts and expression vectors containing overlapping DNA ends were amplified using PCR with appropriately designed primers.

### Buffers

All buffers (Table 1) were prepared fresh the day before the microsome isolation, except the protease inhibitors were added the day of the isolation.

**Table 1.** Composition of the different buffers used in this study.

Buffer	A		B		C	
	50 mM	HEPES-KOH pH 7.6	50 mM	HEPES-KOH pH 7.6	50 mM	HEPES-KOH pH 7.6
	50 mM	KOAc	50 mM	KOAc	250 mM	Sucrose
	6 mM	Mg[OAc]_2_	6 mM	Mg[OAc]_2_	1 mM	DTT
	1 mM	EDTA	1 mM	EDTA	10 µg/ml	Leupeptin^c^
	250 mM	Sucrose	250 mM	Sucrose	1 µg/ml	Chymostatin^b^
	1 mM	DTT	1 mM	DTT		
	0.5 mM	PMSF^a^	0.5 mM	PMSF^a^		
	1 µg/ml	Chymostatin^b^	1 µg/ml	Chymostatin^b^		
	1 µg/ml	Pepstatin^c^	1 µg/ml	Pepstatin^c^		
	10 µg/ml	Leupeptin^c^	10 µg/ml	Leupeptin^c^		
	10 µg/ml	Aprotinin^d^	10 µg/ml	Aprotinin^d^		
			1 µg/ml	Elastatinal^b^		

a Sigma, Saint Louis, MO, USA.

b Merck KGaA, Darmstadt, Germany.

c Thermo Fisher Scientific, Waltham, MA, USA.

d Tebu-Bio, Le Perray-en-Yvelines, France.

### Isolation of microsomes

Canine pancreatic tissues were obtained from a collaborating institution upon completion of animal studies that were conducted in accordance with all institutional and national guidelines for the care and use of laboratory animals.

Dogs (beagle) were sacrificed by intravenous injection of 0.2 mg/kg heparine 5000 IU and sodium pentobarbital (35–48 mg/kg) in the vena cephalica or vena saphena. The dogs were bled by unilateral severance of the vena jugularis and arteria carotis. The pancreas was excised from the animal, immediately snap-frozen and stored at −80°C.

The protocol for the isolation of microsomes from pancreatic tissue is based on the protocol described in Walter and Blobel [23]. Pancreatic tissue was thawed in buffer A (Table 1) on ice. The weight of the pancreatic tissue was determined and the tissue was thoroughly minced with a razor blade and blood vessels and connective tissue were removed, while keeping the pancreatic tissue cold. Three volumes of buffer B (Table 1; according to the weight of the pancreas) were added to the minced tissue. The tissue was extensively homogenized with a tissue homogenizer (TissueRuptor II system, Qiagen) and Dounce Tissue Grinder, while keeping the minced tissue on ice. Tissue homogenate was centrifuged (10 minutes, 1731 g, 4°C) to pellet the cells and nuclei. The pellet was resuspended in one volume of buffer B (Table 1), homogenized in the douncer and centrifuged (10 min, 1731 g, 4°C). The supernatant of both fractions was sieved through a cell strainer (Corning, 40 µm pore) and centrifuged (Ti-45 rotor, Sorvall WX Ultra 80, 18 min, 10 037 g, 4°C) to separate Golgi, mitochondria, peroxisomes and lysosomes from the microsomes. The supernatant was sieved and layered on top of the sucrose cushion (50 mM HEPES-KOH pH 7.6, 50 mM KOAc, 6 mM Mg[OAc]_2_, 1 mM EDTA, 1300 mM sucrose, 1 mM DTT, 1 µg/ml chymostatin, 1 µg/ml pepstatin, 10 µg/ml leupeptin, 10 µg/ml aprotinin, and 1 µg/ml elastatinal) (ratio sample to cushion was ∼3:1). After ultracentrifugation (Ti-45 rotor, Sorvall WX Ultra 80, 3.5 h, 235 000 g, 4°C) the supernatant was removed by aspiration. The membrane pellet was resuspended in buffer C (Table 1) and homogenized thoroughly in the Dounce Tissue Grinder. The absorbance at 280 nm (A_280_) and the ratio A_260_/A_280_ was measured (in 1% (w/v) sodium dodecyl sulphate (SDS) solution) using the Nanophotometer N60 (Implen). The concentration (equivalents (eq)/µl) was calculated according to the formula described in [23]. Membrane preparations were snap-frozen in liquid nitrogen and stored at −80°C until further use.

### Cell-free *in vitro* translation and translocation

The DNA of interest from the pGEM4 plasmids was amplified and linearized by using PCR (HotStart Taq DNA polymerase, New England Biolabs) and PCR products were purified with the nucleospin gel and PCR clean-up kit (Machery Nagel). DNA of interest was then transcribed *in vitro* using T7 RNA polymerase (RiboMAX system, Promega) and RNA was purified using the Nucleospin RNA clean-up kit (Machery Nagel). mRNA transcripts were translated in a cell-free system with rabbit reticulocyte lysate (RRL) (Promega) in the presence of L-[^35^S]-methionine (Hartmann analytic GmbH). Translations were performed at 30°C for 45 min, in the presence or absence of canine pancreatic microsomes and CADA (or 1% DMSO as negative control), supplemented with RNase inhibitor RNasin (Promega). Proteinase K (PK, Roche) digestion experiments were performed (253 ng/µl PK) on ice for 30 minutes and quenched with 10 mM PMSF on ice. Samples were applied on sucrose cushion (50 mM HEPES-KOH pH 7.5, 10 mM Mg[OAc]_2_, 200 mM KOAc, 1 mM DTT and 0.5 M sucrose) and centrifuged for 30 min at 436 000 g (TLA100 rotor, 4°C)(Optima MAX-XP ultracentrifuge, Beckman Coulter, RRID: SCR_025704). Pellets were dissolved in reducing SDS sample buffer (120 mM Tris–HCl pH 6.8, 4% (w/v) SDS, 20% (v/v) glycerol, 100 mM DTT, and 0.02% (w/v) bromophenol blue) and analyzed by SDS-polyacrylamide gel electrophoresis (SDS-PAGE) on 4–12% NuPage Bis-Tris Midi Protein gels (Invitrogen) in NuPage MES SDS running buffer (Invitrogen). For increased separation of translocated protein species from the full length preprotein, samples were analyzed by hand-cast polyacrylamide gels consisting of a 4% (v/v) stacking (125 mM Tris/pH 6.8, 4% (w/v) acrylamide-, bis-acrylamide solution (37.5:1) (BioRad), 0.1% (w/v) SDS, 0.05% (v/v) tetramethylethylenediamine (TEMED), 0.05% (w/v) ammonium persulphate (APS)) and a 12.5% (v/v) separating (375 mM Tris/pH 8.8, 12.5% (w/v) acrylamide-, bis-acrylamide solution (37.5:1), 0.1% (w/v) SDS, 0.05% (v/v) TEMED, 0.05% (w/v) APS). The polyacrylamide gels were dried using the model 582 gel dryer (BioRad) and HydroTech Vacuum pump (BioRad). Radioactive-labeled proteins were detected by phosphor imaging (Typhoon FLA 9500 imager, GE Healthcare Life Sciences, RRID: SCR_019957), and quantified using the ImageQuant software (Cytiva, RRID: SCR_018374).

### Immunoblotting

Dog microsomes were diluted in reducing SDS sample buffer to a concentration of 1 eq and heated for 10 min at 80°C. The samples were loaded on a 10% NuPage Bis–Tris Midi Protein gel (Invitrogen) in NuPage MES SDS running buffer (Invitrogen) and transferred to a polyvinylidene difluoride (PVDF) membrane (BioRad) using the BioRad Trans-Blot Turbo transfer system (BioRad). After blocking with 5% (w/v) nonfat dried milk in Tris-buffered saline with Tween 20 (TBST) (137 mM NaCl, 20 mM Tris–HCl pH 7.6, 0.05% (v/v) Tween 20), the PVDF membrane was incubated with a primary antibody (Table 2) O/N at 4°C on a rocking platform. Membranes were washed with TBST and incubated with the secondary antibody (Table 2) for 1 h at room temperature. Membranes were washed again with TBST to reduce background staining. Protein detection was performed with SuperSignal West Pico and Femto chemiluminescence reagent (ThermoFisher) and the ChemiDocMP Imaging System (BioRad, RRID: SCR_019037). Signal intensities were quantified with Image Lab software, version 5.0 (BioRad, RRID: SCR_014210).

**Table 2.** Overview of the antibodies used for immunoblotting.

Antibody	Source	Identifier (Cat#, RRID)
Sec61α	Cell Signaling Technology^a^	Cat #14867, AB_2798635
Sec61β	Gift from collaborator^b^	/
TRAP	Gift from collaborator^b^	/
BiP	BD Biosciences^c^	Cat #610979, AB_398292
RPL26	Cell Signaling Technology^a^	Cat #5400, AB_10698750
HRP goat anti-mouse IgG	Dako^d^	Cat #P0447, AB_2617137
HRP swine anti-rabbit IgG	Dako^d^	Cat #P0399, AB_2617141

a Cell Signaling Technology, Danvers, MA, USA.

b Prof. Kalies, Centre for Structural and Cell Biology in Medicine, Institute of Biology, University of Lübeck, Lübeck, Germany.

c BD Biosciences, San Jose, CA, USA.

d Dako, Santa Clara, CA, USA.

## Results

### Isolation of microsomes from snap-frozen canine pancreatic tissue

Rough microsomes (RM) isolated from animal pancreatic tissue are the common source of ER membranes for cell-free *in vitro* protein translation and translocation studies [12]. The original protocol for the extraction of microsomes from fresh tissue has been described several decades ago by Walter and Blobel [23]. In this study, we explored if snap-freezing and defrosting of the pancreatic tissue after storage at −80°C would be detrimental to the functionality of the isolated microsomes.

Pancreatic tissues from 20 Beagle dogs were obtained from a collaborating institution upon completion of animal studies at different time points between October 2022 and October 2023. At end-points of these clinical studies, animals were euthanized for subsequent histological examination. In parallel, pancreatic tissue that would have been discarded, was excised from the dogs, immediately snap-frozen and stored at −80°C until downstream processing. After thawing of the tissue on ice, rough microsomes were extracted according to the protocol described in the Materials & Methods section and the microsome pellet was dissolved in buffer C (Table 1) for storage at −80°C. The final microsome concentration, reported as “equivalent (eq),” for each pancreas was calculated based on the formula proposed by Walter and Blobel [23]. As summarized in Table 3, for three samples (#06, #08, and #11) no microsome pellet could be recovered after the final ultracentrifugation step and these samples were discarded. However, for the remaining 17 samples, extraction of the microsomes was successful with good yield and microsome density. Of note, the different amounts of adipose tissue in the defrosted weighted sample (removed at the start of the extraction) can partially explain the variation in the microsome yield relative to the initial amount of pancreas (Table 3).

**Table 3.** Characteristics of the microsome samples.

Dog ID number	Weight pancreas net (mg)^a^	Weight adipose tissue (mg)^b^	Amount RM isolated (eq)	Ratio RM/pancreas (eq/mg)	RM concentration (eq/µl)	Purity (A_260_/A_280_)
01	8300	ND^c^	13 500	1.63	2.7	1.83–1.87
02	20 500	ND	28 000	1.37	4	1.87
03	24 900	ND	42 000	1.69	2.8	1.75–1.81
04	20 800	11 500 (36%)	16 500	0.79	3.3	1.86–1.89
05	19 500	6800 (26%)	16 000	0.82	2	1.87–1.88
06	21 700	8000 (27%)	NP^d^			
07	25 300	8200 (24%)	95 000	3.75	3.8	1.78
08	6500	3100 (32%)	NP			
09	26 500	2500 (9%)	80 920	3.05	2.89	1.83–1.86
10	16 800	1800 (10%)	17 400	1.03	2.9	1.83
11	28 700	4900 (15%)	NP			
12	22 400	6000 (21%)	29 500	1.32	2.95	1.83
13	27 400	3800 (12%)	79 640	2.91	3.62	1.82–1.84
14	22 200	3500 (14%)	66 000	2.97	4.4	1.82–1.83
15	21 400	3100 (13%)	48 720	2.28	3.48	1.86
16	9000	1500 (14%)	26 200	2.91	2.62	1.82–1.83
17	11 200	1300 (10%)	35 900	3.21	3.59	1.77–1.79
18	26 700	2100 (7%)	114 000	4.27	4.56	1.80–1.82
19	19 000	2300 (11%)	45 240	2.38	3.48	1.85–1.87
20	24 000	900 (4%)	59 520	2.48	3.72	1.82–1.85

a Net weight pancreas after removal adipose tissue.

b Amount of adipose tissue removed from original pancreatic tissue. The value between brackets indicates the amount of adipose tissue relative to the total weight of the initial pancreatic tissue.

c ND, not determined; data not available.

d NP, no pellet.

### Identification of ER translocon subunits and associating factors in rough microsomes purified from frozen pancreatic tissue

**Figure 1:**
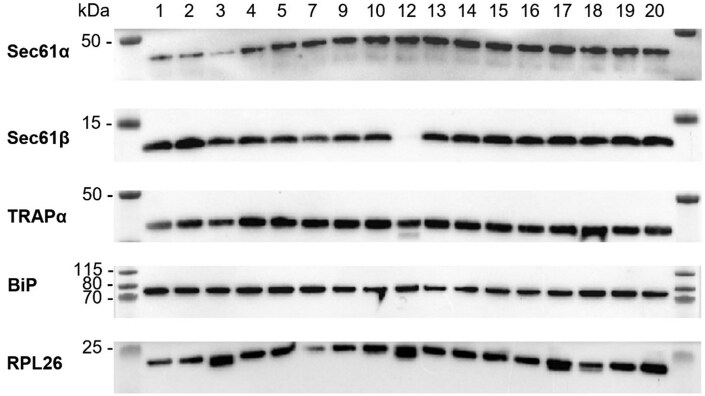
Extracted rough microsomes (RM) from snap-frozen pancreatic tissue contain ER translocon subunits and associating factors. Western blot analysis of Sec61 translocon subunits and ER translocation-associating factors in the extracted RM samples from 17 different dogs. Selected proteins were identified by western blotting with conditions optimized for each individual primary antibody. Representative images are shown for the following proteins: α subunit of the Sec61 translocon (predicted MW 52 kDa), β subunit of the Sec61 translocon (MW 10 kDa), α subunit of TRAP (MW 32 kDa), BiP (MW 72 kDa), and 60S ribosomal protein L26 (RPL26; MW 17 kDa). The numbers at the top of the figure refer to the ID of each dog sample as in Table 3. The first and last lanes show the MW marker (in kDa).

To assess if the isolated membrane fractions contain ER-derived microsomes, we performed a detailed western blot analysis on 17 membrane samples. As shown in Fig. 1, all samples contained detectable amounts of Sec61α, the pore-forming subunit of the heterotrimeric Sec61 translocon complex, which is essential for protein translocation. The smaller peripheral subunit Sec61β of the complex was detected in all microsome batches, except in sample #12. In addition, TRAPα, a translocating co-factor of the ER i.e. associated with Sec61, was clearly present in all of our microsome samples. Also, the lumenal factor BiP was identified in all of our samples, indicating that the microsomes were intact (and not leaky) with preserved lumenal content. Finally, ribosomal protein L26 (RPL26), a component of the 60S subunit of ribosomes (that are attached to the ER translocon), was also detected in all of our isolated RM fractions. All together, these data indicate that several of the subunits and chaperones involved in protein translocation across the ER membrane are present in our microsome samples extracted from snap-frozen pancreatic tissue.

### Microsomes isolated from frozen pancreatic tissue are translocation competent and contain signal peptidases with preserved proteolytic activity

**Figure 2:**
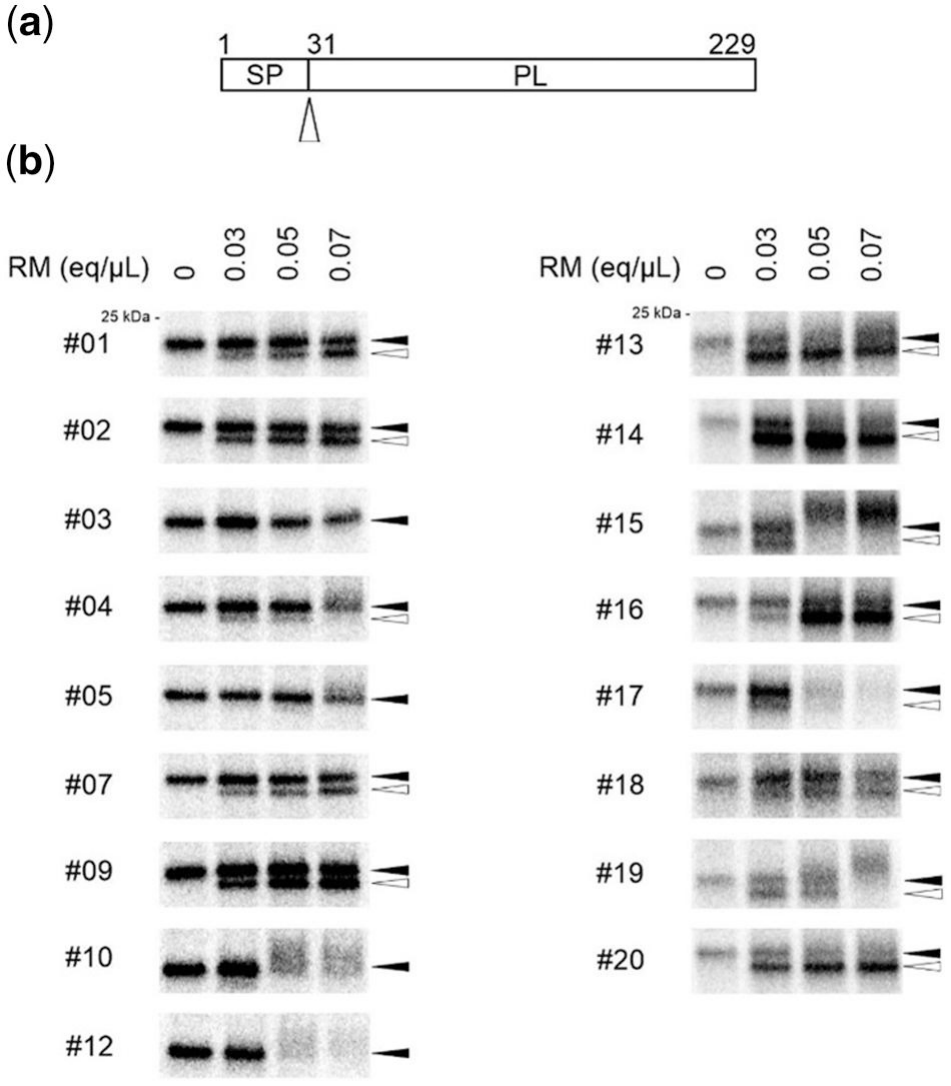
Translocation of preprolactin in extracted rough microsomes (RM) from snap-frozen pancreatic tissue. (a) Schematic representation of preprolactin (pPL) with its signal peptide (SP) i.e. cleaved by the ER lumenal signal peptidase generating mature prolactin (PL). The cleavage site is indicated with an open arrowhead. (b) Cell-free *in vitro* translation and translocation reactions were performed using rabbit reticulocyte lysate supplemented with increasing concentrations (0.03, 0.05, or 0.07 eq/µl) of RMs from each dog sample and compared to a condition in the absence of RM (first lane; “0”). In the presence of translocation competent RM, bovine pPL (solid arrowhead) is translocated into the ER lumen and SP-cleaved, resulting in a faster migrating mature protein (open arrowhead). Representative autoradiograms are shown. The numbers at the left of each autoradiogram refer to the ID of the respective dog sample listed in Table 3. Note that the pPL signal in samples #13–20 is weaker as compared to samples #1–12 because of a different experimental setting (other prep of RNA and more radioactive decay of S^35^ methionine).

In order to investigate the functionality of the isolated microsomes, we first assessed the translocation of wild-type bovine preprolactin (pPL), a prototype secretory protein that, in general, gets translocated into the ER lumen with high efficiency. During co-translational translocation of nascent polypeptide chains through the Sec61 translocon channel, the N-terminal signal peptide is cleaved from the preprotein by a signal peptidase located in the lumen of the ER (Fig. 2a). This proteolytic cleavage gives rise to a mature protein with a slightly lower molecular weight (MW) that will appear as a faster migrating band on the gel as compared to the preprotein (Fig. 2b; open and solid arrowhead, respectively). A balanced amount of microsomes is needed in order to achieve maximum translocation (i.e. insufficient amount of microsomes results in little translocation; excessive amount of microsomes slows down protein translation by the ribosomes). Therefore, initial testing of each dog RM sample was performed with three different concentrations of microsomes (i.e. 0.03, 0.05, and 0.07 eq/µl), and compared to a condition of pPL translation in the absence of RM (“0”). As shown in Fig. 2b, in 13 out of the 17 RM samples (i.e. #1, 2, 4, 7, 9, 13, 14, 15, 16, 17, 18, 19, and 20), detectable translocation of pPL was obtained at the lowest RM input, as determined by the presence of SP-cleaved species. Whereas for most of our samples, a higher microsome concentration correlated with elevated levels of translocated protein, increasing the RM concentration of samples #4, 15, 17, and 19 seemed to negatively affect pPL translation. For the remaining four RM samples (i.e. #3, 5, 10, and 12) no SP-cleaved species could be visualized at the lowest RM concentration, and increasing the amount of RM in the translation reaction mixture was detrimental for protein translation. Of note, for one of the inactive RM samples (#12) no Sec61β subunit was detected with Western Blot (Fig. 1).

**Figure 3:**
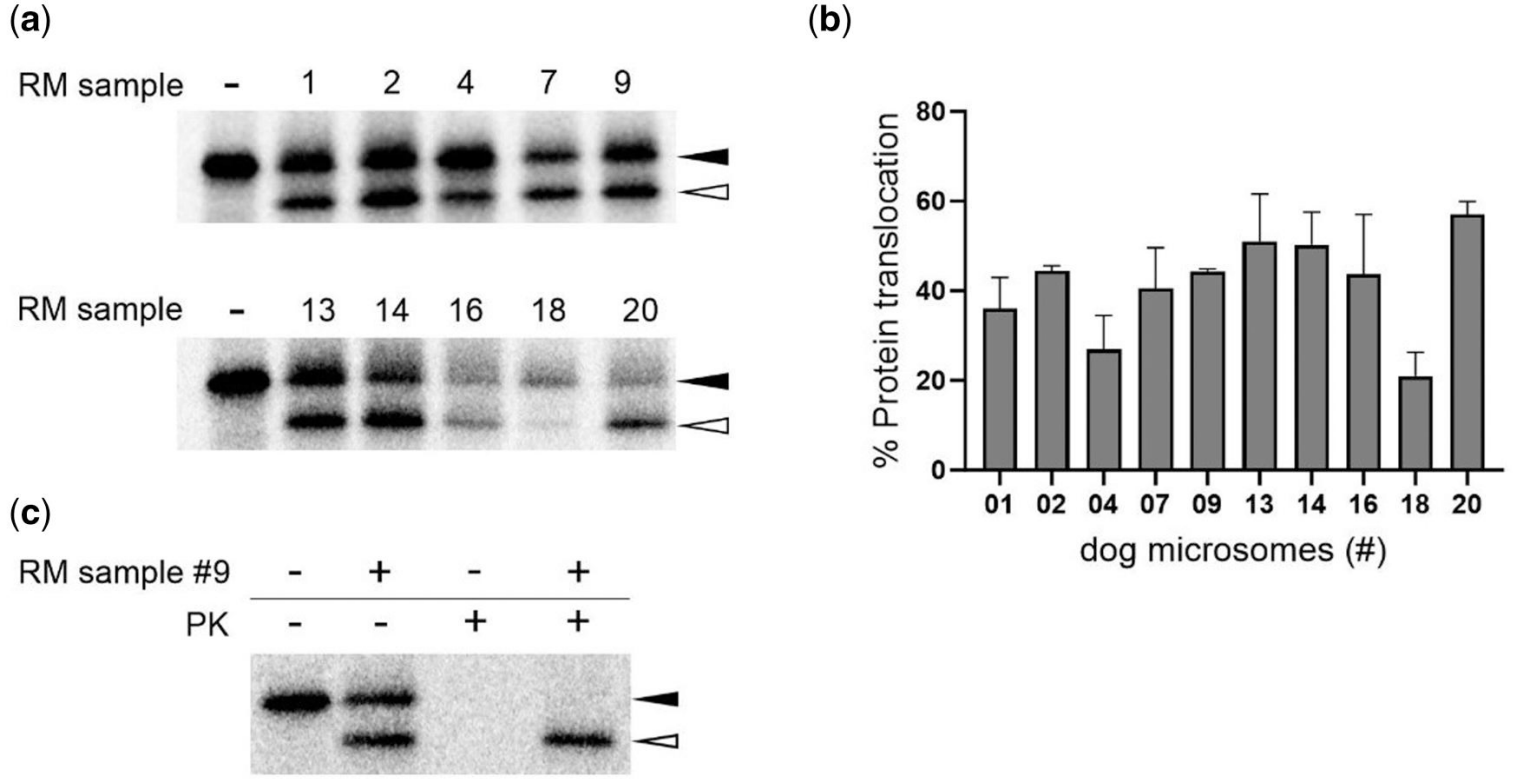
Translocation efficiencies for preprolactin (pPL) in extracted rough microsomes (RM) from snap-frozen pancreatic tissue. (a) Autoradiogram of cell-free *in vitro* translated and translocated prolactin in the presence of optimized RM concentrations for a selection of 10 dog samples. The first lane shows a condition of pPL translation in the absence of RM (“-”). Solid arrowhead = pPL precursor; open arrowhead = SP-cleaved prolactin. One representative experiment out of three is shown. The numbers at the top of each autoradiogram refer to the ID of the respective dog sample listed in Table 3. (b) Quantification of the translocation efficiency of pPL from three independent experiments shown in (a). For each dog sample, the percentage of protein translocation is calculated based on the signal intensity for the protein band of the cleaved species relative to the total amount of protein (SP-cleaved + precursor) and normalized to background noise signal. Bars represent mean ± SD; *n = *3. (c) PK assessment. Autoradiogram of cell-free *in vitro* translated and translocated prolactin in the presence of dog RM#9 (0.07 eq/µl), supplemented with PK. Solid arrowhead = pPL precursor; open arrowhead = SP-cleaved prolactin.

Based on this initial analysis, we selected the optimal RM concentration of 10 dog samples for further characterization of the extracted microsomes. First, the translocation activity of each RM sample was quantified for the translocation of pPL in three independent experiments. To obtain a better separation of the SP-cleaved species from the full length preprotein, samples were run on large 12.5% TRIS–HCl gels. As summarized in Fig. 3, the translocation efficiency for pPL varied between 21% (sample #18) and 57% (sample #20) as determined by the fraction of SP-cleaved species (open arrowhead) relative to the sum of SP-cleaved and preprotein fraction (open + solid arrowhead). Interestingly, for 7 out of 10 RM samples, a translocation efficiency of at least 40% was obtained. To verify that the faster migrating protein band on the gel (open arrowhead) is the SP-cleaved protein i.e. fully translocated into the ER lumen, an additional proteinase protection assay was performed. Briefly, protein samples are being exposed to PK which will digest all protein species in our sample, except those that are in the protected environment of the ER lumen. As shown for a representative sample (#9) in Fig. 3c, the SP-cleaved species are fully resistant to PK. This also confirms that the extracted microsomes are not leaky.

### Microsomes isolated from frozen pancreatic tissue contain translocon-associated OST complexes with preserved glycosylation activity

**Figure 4:**
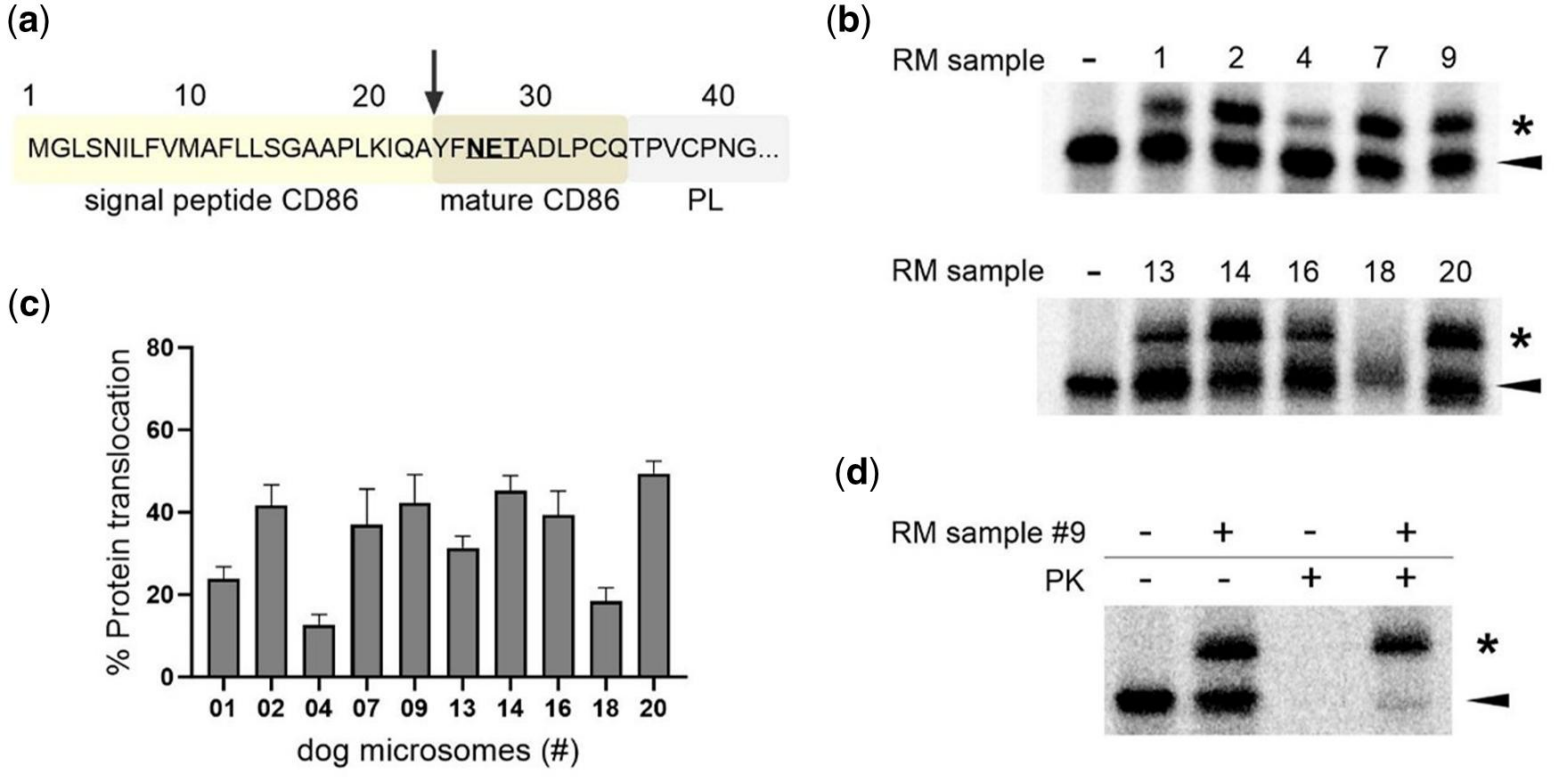
Glycosylation efficiency of CD86 reporter in extracted rough microsomes (RM) from snap-frozen pancreatic tissue. (a) Schematic representation of the CD86-prolactin (PL) chimeric reporter composed of the 24-residue signal peptide and 11 residues of the mature human CD86 protein fused to the N-terminal part of mature bovine PL. The vertical black arrow indicates the signal peptide cleavage site. The short N-terminal stretch of mature CD86 contains the N-glycosylation site (depicted in bold and underlined). (b) Autoradiogram of cell-free *in vitro* translated and translocated CD86-PL chimeric protein in the presence of optimized RM concentrations for a selection of 10 dog samples. The first lane shows a condition of protein translation in the absence of RM (“-”). Solid arrowhead = CD86-PL precursor; asterisk = glycosylated protein. One representative experiment out of three is shown. The numbers at the top of each autoradiogram refer to the ID of the respective dog sample listed in Table 3. (c) Quantification of the glycosylation efficiency of CD86-PL from three independent experiments shown in (b). For each dog sample, the percentage of protein translocation is calculated based on the signal intensity for the protein band of the glycosylated species (_*_) relative to the total amount of protein (glycosylated + precursor) and normalized to background noise signal. Bars represent mean ± SD; *n = *3. (d) PK assessment. Autoradiogram of cell-free *in vitro* translated and translocated CD86-PL chimeric protein in the presence of dog RM#9 (0.07 eq/µl), supplemented with PK. Solid arrowhead = CD86-PL precursor; asterisk = glycosylated protein. Note that a very small fraction of translocated protein does not get glycosylated (last lane; solid arrowhead).

In addition to signal peptide cleavage, co-translational translocation of nascent polypeptide chains coincides with N-glycosylation of proteins in the ER lumen. Translocon-associated OST complexes will add sugar moieties to asparagine residues at specific N-glycosylation sequons. Given that this protein modification process in the ER lumen occurs during translocation of the protein, glycosylation of the substrate can be used as a parameter of translocation efficiency into the ER. To this end, a glycosylation reporter substrate was evaluated, as reported earlier [13]. More specifically, we used a construct in which the SP and first 11 amino acid residues of the mature protein of human CD86 were fused to the N-terminus of mature prolactin (PL; Fig. 4a). This construct contains only one glycosylation sequon located at the short N-terminal stretch of mature CD86. Translocation of this reporter construct will give rise to SP-cleaved species that are glycosylated, resulting in a translocated protein with a net increase in MW (Fig. 4b; asterisk) as compared to the preprotein (solid arrowhead). As summarized in Fig. 4c, all tested RM samples showed glycosylation of the CD86 reporter, although with varying efficiency ranging from 10% to 52%. Remarkably, samples #4 and #18 that showed the lowest levels of pPL translocation expressed the lowest glycosylation efficiencies, whereas sample #20 performed best for both SP-cleavage and glycosylation. An additional proteinase protection assay on a representative sample (#9) confirmed that the glycosylated species are fully translocated and reside in the lumen of the ER (Fig. 4d).

### Isolated microsomes from frozen pancreatic tissue preserved their sensitivity to the translocation inhibitor CADA

**Figure 5:**
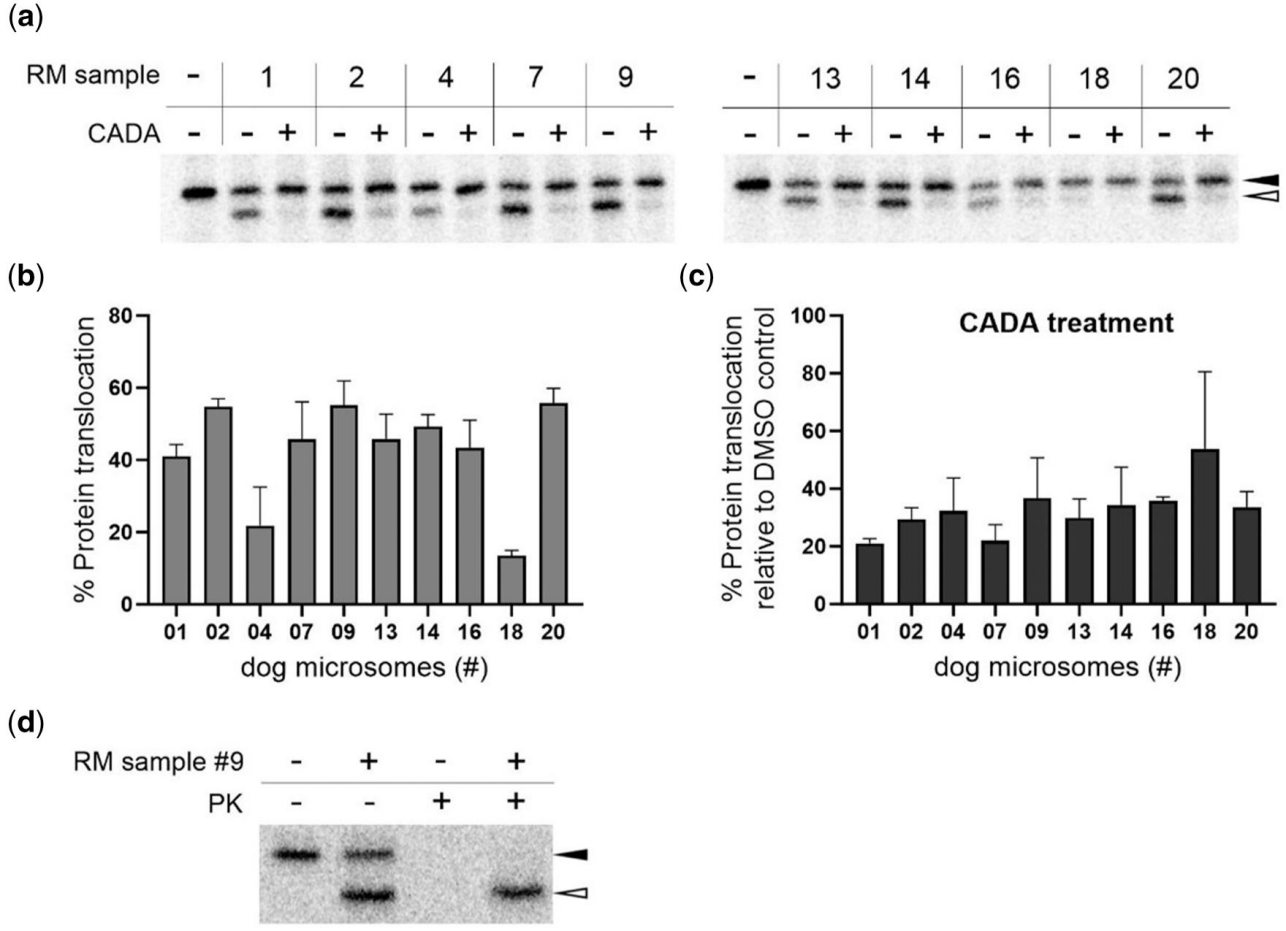
CD4 translocation inhibition by CADA in extracted rough microsomes (RM) from snap-frozen pancreatic tissue. (a) Autoradiogram of cell-free *in vitro* translated and translocated CD4 (truncated construct containing only N-terminal domains D1 and D2) in the presence of optimized RM concentrations for a selection of 10 dog samples. The first lane shows a condition of protein translation in the absence of RM (“-”). For each RM sample, microsomes were treated either with solvent control DMSO (“-”) or with CADA (150 µM). Solid arrowhead = CD4 precursor; open arrowhead = SP-cleaved CD4. One representative experiment out of three is shown. The numbers at the top of each autoradiogram refer to the ID of the respective dog sample listed in Table 3. (b) Quantification of the percentage of CD4 translocation in the DMSO controls, calculated from the signal intensities in (a). Values are means ± SD; *n = *3. (c) Quantification of the translocation efficiency of CD4 in the presence of RM and CADA, intensities calculated from (a). For each dog sample, the amount of CD4 translocation in the presence of CADA was normalized to CD4 translocation of the respective DMSO control (set as 100%). Values are means ± SD; *n = *3. (d) PK assessment. Autoradiogram of cell-free *in vitro* translated and translocated CD4 in the presence of dog RM#9 (0.07 eq/µl), supplemented with PK. Solid arrowhead = CD4 precursor; open arrowhead = SP-cleaved CD4.

Finally, to complete the characterization of the extracted microsomes, we also assessed the sensitivity of the RM samples to a translocation inhibitor. As previously reported, the small-molecule CADA is a Sec61 inhibitor with high substrate selectivity for human CD4 [24, 25]. For an easy read-out of our translocation assay, we made use of a truncated form of the membrane-anchored CD4 receptor, missing the C-terminal D3 and D4 immunoglobulin-like domains and the transmembrane domain, as described in an earlier study [22]. Similar to pPL, translocation of this truncated CD4 construct results in a SP-cleaved mature protein (open arrowhead) that migrates faster on the gel as compared to the preprotein (solid arrowhead). Again, all 10 dog RM samples were tested for CD4 translocation, both in the absence (i.e. DMSO control) or presence of the inhibitor CADA. As shown in Fig. 5a, for all of our tested samples, in the absence of CADA detectable SP-cleavage of CD4 was obtained, although in varying efficiency (Fig. 5b). Interestingly, the variation in the translocation efficiency observed for CD4 in the different RM samples resembled well to what was obtained for pPL. More specifically, microsomes from sample #20 performed best (56% translocation), whereas those from samples #4 and #18 exerted the lowest SP-cleavage activity (22% and 12%, respectively). As expected, addition of CADA resulted in a clear suppressive effect on the translocation of CD4 in all tested RM samples (Fig. 5c). Similar to pPL, an additional proteinase protection assay on a representative sample (#9) confirmed that the SP-cleaved species are fully translocated and reside in the lumen of the ER (Fig. 5d). Altogether, these data demonstrate that microsomes isolated from frozen pancreatic tissue retain their translocation competence and respond equally well to a translocation inhibitor.

## Discussion

Microsomal membranes isolated from pancreatic tissue of dogs serve as the primary source of ER membranes to study cell-free *in vitro* protein translation and translocation into the ER. Although pancreatic microsomes from other animals have been explored, microsomes from dogs outperform those from sheep and pigs as they are highly compatible with *in vitro* translation systems such as rabbit reticulocyte lysate [12, 16, 26]. However, ethical questions and the high costs make it extremely challenging to justify sacrificing healthy dogs solely for the isolation of the pancreas. Here, we demonstrate that snap-frozen canine pancreatic tissue (from sacrificed beagle dogs at end-points in clinical studies) can serve as an alternative to freshly isolated pancreatic tissue for the extraction of microsomes, thereby reducing animal use by utilizing tissue from dogs sacrificed for other purposes.

Freezing and thawing of organ tissue can cause severe damage to the cells which can result in the release of several cellular proteases that can degrade essential proteins in the *ex vivo* sample. Moreover, pancreatic tissue actively secretes digestive enzymes, making it crucial to reduce the time between sacrificing of the dogs and the actual extraction of the microsomes from the tissue as reported in the original isolation protocol [23]. By maintaining the defrosted sample on ice during the extraction procedure in the presence of a suitable buffer that contained several protease inhibitors, we managed to extract microsomes that comprised the necessary translocon components and chaperones as verified by immunoblotting (Fig. 1), such as the Sec61α and Sec61β translocon core components and associated factor TRAP, consistent with reports for fresh canine microsomes [16, 27]. However, immunoblotting does not provide information about the native state and integrity of those translocon subunit protein complexes. In one of our microsome samples, Sec61β was not present at detectable levels and those microsomes failed to translocate bovine preprolactin. However, in the three other RM samples that were unsuccessful in protein translocation, Sec61β was clearly detected, indicating that the presence or absence of this small translocon subunit is not a predictive marker for translocation functionality of our RM samples. This is in line with a report that demonstrated that Sec61β kinetically favors co-translational translocation of clients but is not essential for channel functioning [10, 28]. The inactivity of the other RM samples might be related to e.g. protease leakage during thawing that degrade part of the translocon that interacts with the ribosome, ribosome detachment, depletion of essential co-factors for translocation such as SRP and its receptor, impurities or excess of lipid debris. However, a clear explanation is hard to formulate given that all remains very speculative as different (unknown) factors might contribute to optimal protein translocation.

Another important characteristic of the RM sample is the integrity of the microsome membrane. Preservation-induced cold ischemia can cause pancreatic edema and enzyme leakage, which depends on the membrane motility and sample quality at the moment of freezing. Therefore, a cryoprotectant, such as a high sucrose buffer, is added during freezing to preserve the membrane structure and function. Given that a freeze-thaw cycle might disrupt the lipid bilayer we next verified the presence of BiP in our RM samples as this is a soluble chaperone that resides in the lumen of the ER and would exit the ER if the membrane was being permeabilized [29]. In addition, resistance of the translocated proteins to PK exposure provided solid proof of the integrity of the microsome membrane.

Through cell free *in vitro* translation/translocation experiments we could demonstrate detectable translocation of pPL in 13 out of the 17 RM samples and validate the functionality of the microsomes. Furthermore, in 80% of the tested RM samples, a productive translocation (>40%) was achieved for CD4 (Fig. 5b), a reporter protein with a rather weak SP (as compared to pPL) [24]. Whereas for CD4 comparable translocation was obtained with our RM samples as compared to what we reported in earlier studies [22, 24], the translocation efficiency for pPL with our RM samples was lower as what is generally seen by others (60–80%) [23]. However, in our hands we never reached translocation levels for pPL that exceeded 60% [22, 24], which might be related to the type of pPL expression plasmid and/or mRNA preparation/purification and the quality of the transcripts. Importantly, the competent microsome samples also supported efficient glycosylation of the translocated clients, indicating that the microsomes express a functional OST complex. The validated microsome samples showed some inter-sample variability but returned a consistent translocation activity over the different substrates, with sample #20 identified as being the best performer, and samples #4 and #18 the ones with weak activity. The variation in functionality between the RM samples could not be strictly linked to the adipose content of the pancreatic tissue (Table 3). Although a higher amount of adipose tissue seems to negatively correlate with the relative amount of purified microsomes for the sample with the highest adipose content (e.g. sample #04), this seems not to be a general pattern (compare #07 with #19). Notably, our best performer (RM #20) had the lowest amount of adipose tissue (4%, Table 3), however, the content of adipose tissue in the pancreas seemed not to be a general predictor for functionality of the RM. Two samples (#09 and #18) with relative equal low amounts of adipose tissue had a 2-fold difference in translocation activity for pPL (44 and 21%, respectively). Finally, CD4 translocation was effectively blocked by the inhibitor CADA in all of the tested microsomes, indicating that these RM samples can be implemented in future studies on Sec61 translocon inhibitors to explore the therapeutic potential of such inhibitors in the field of oncology, immunology and microbiology [30].

Our data proved full functionality of the extracted ER microsomes with preserved translocation competence. Therefore, we consider our adapted protocol for the extraction of microsomes from defrosted pancreatic tissue as being a successful alternative to the original one. Having the option to snap-freeze the pancreas samples offers the advantage to physically and temporally spread the excision of the tissue and the actual performing of the microsome extraction, thus, controlling the workload. *In vitro* translocation experiments can alternatively be performed with semipermeabilized cells from different cell cultures (such as human HeLa or PC3 cells), however, only small amounts of microsomes can be obtained and the experimental setting requires fresh cells, which might challenge the reproducibility of the experiments [31, 32]. Extraction of large amounts of microsomes from canine pancreatic tissue will remain essential to perform additional structural studies, such as Cryo-EM analysis of Sec61 complexes, as this technique relies on a homogenous source of microsomes in sufficient amounts that can only be derived from animal tissue.

In conclusion, we demonstrated that snap-frozen canine pancreatic tissue can serve as an alternative source to fresh pancreatic tissue for the isolation of translocation competent microsomes that can be applied in cell-free *in vitro* protein translocation studies.

## Data Availability

Data available on request: the data underlying this article will be shared on reasonable request to the corresponding author.
